# *TREML4* polymorphisms increase the mRNA in blood leukocytes in the progression of atherosclerosis

**DOI:** 10.1038/s41598-022-22040-3

**Published:** 2022-11-03

**Authors:** Victor Hugo Rezende Duarte, Marina Sampaio Cruz, Adriana Bertolami, Mario Hiroyuki Hirata, Rosario Dominguez Crespo Hirata, André Ducati Luchessi, Vivian Nogueira Silbiger

**Affiliations:** 1 Department of Clinical and Toxicological Analyses, Federal University of Rio Grande do Norte, Avenue General Gustavo Cordeiro de Farias, S/N, Natal, Rio Grande do Norte 59012-570 Brazil; 2 Dyslipidemia Medical Section, Dante Pazzanese Institute of Cardiology, Av. Dr. Dante Pazzanese, 500, São Paulo, 04012-909 Brazil; 3 Department of Clinical and Toxicological Analyses, School of Pharmaceutical Sciences, University of Sao Paulo, 580 B17 Lineu Prestes Av., Butantan, São Paulo, 05508-900 Brazil

**Keywords:** Genetics, Molecular biology, Biomarkers, Cardiology

## Abstract

*TREML4* and other members of the triggering receptor expressed in the myeloid cell family are associated with a risk of atherosclerosis and progression in coronary artery disease, acute coronary syndrome, and coronary artery calcification. Herein, the relationship between *TREML4* expression and its polymorphisms (rs2803495 and rs280396) was evaluated in patients with subclinical atherosclerosis (n = 340) and heart failure post-acute myocardial infarction (MI) (n = 68) for the first time. *TREML4* variants rs2803495 (A > G) and rs2803496 (T > C) and leukocyte mRNA expression was analyzed by qRT–PCR. The rs2803495 G allele was associated with *TREML4* expression (OR 8.01, CI 3.78–16.99, *p* < 0.001). Patients carrying the rs2803496 C minor allele (TC/CC genotypes) were more likely to express *TREML4* than those without the C allele (OR 10.42, CI 4.76–22.78, *p* < 0.001), as well as having higher levels of TREML4 expression (OR 4.88, CI 2.35–10.12, *p* < 0.001). Thus, we report for the first time that TREML4 is not associated with the early stages of atherosclerotic plaque formation and later stages after MI. In conclusion, TREML4 mRNA expression in blood leukocytes is influenced by minor alleles (G and C) and may regulate differently during the atherosclerosis progression stages, but not in asymptomatic atherosclerosis disease and post-MI.

## Introduction

Cardiovascular diseases are common, have high lethality, and are increasing worldwide^1^. Atherosclerosis is a progressive disease with a prolonged latent phase and clinically unapparent lesions, so its acute or chronic manifestations demonstrate a more significant atherosclerotic burden or more advanced stages of the disease^2^.

The atherosclerotic process begins with the migration and retention of lipids, especially LDL cholesterol, recruitment of inflammatory cells, and formation of foam cells with subsequent apoptosis, necrosis, calcification, and arterial remodeling, which may result in plaque inflammation, rupture, thrombosis, and other cardiovascular diseases. Due to the complex interaction between these processes, most plaques remain asymptomatic (subclinical atherosclerosis, SA); others become obstructive (stable angina) or even cause acute thrombosis leading to acute coronary syndrome (ACS)^3^. The ACS range from unstable angina to non–ST-segment elevation myocardial infarction (MI), ST-segment elevation myocardial infarction, and sudden cardiac death. The consequence of extensive myocardial damage and impairment of myocardial contractility, modifying the left ventricular (LV) structure, in a process known as LV remodeling (LVR), which reduce LV function^4^.

Genome-wide association studies have identified 164 gene loci that confer an increased risk of coronary artery disease and Atherosclerosis, and the role of many of these genes and pathways has not yet been adequately investigated^5^. However, many genes are already well described in the literature as being associated with the development of Atherosclerosis, such as genes for lipid metabolism (*APOA5, LDLR, APOB*), platelets and blood coagulation (*PCSK9, TGFB1*), and cell migration and adhesion (*NCK, IRS1*)^5^. Furthermore, new genes have shown a promising role in developing cardiovascular diseases in the last decade, such as *TREML4*^6–8^*.*

*TREML4* is a member of the Triggering receptor family expressed on myeloid cells (TREM). It is a single immunoglobulin (IgV) variable, with domain activating receptors on the surface of neutrophils/monocytes and dendritic cells^9^. The Triggering Receptor Expressed on Myeloid cells-Like 4 (*TREML4*) belongs to the triggering receptor expressed on the myeloid cell (*TREM*) family located on chromosome 6, which is composed of receptors of a single extracellular variable-type immunoglobulin-like domain and are structurally similar^9^. In previous studies, we showed that the expression of *TREML4* and its polymorphisms (rs2803495 and rs2803496) are associated with different clinical outcomes of atherosclerosis, such as coronary artery disease^6^, ACS^7^, and Coronary Artery Calcium (CAC)^8^. Based on our previous results, *TREML4* is likely involved in the atherosclerotic progress to plaque disruption ending up in MI and might be a potential biomarker. However, no studies have investigated its relationship with stages that precede or succeed atherosclerotic progress to MI and after the rupture in heart failure post-MI.

We previously investigated the relationship between gene expression and TREML4 polymorphisms in patients with coronary artery disease undergoing coronary angiography. We demonstrated that TREML4 mRNA expression in leukocytes is influenced by the extent of coronary artery lesions and genetic polymorphisms (RS2803495 and rs2803496), so the more significant the atherosclerotic burden, the greater the expression of TREML4^6^. In 2013, we were the first group to demonstrate the relationship between *TREML4* expression and patients with ACS. Through transcriptomic analysis by microarray technology, we demonstrated that the differential expression of *TREML4* is related to the early stages of ACS and that *TREML4* can be used to monitor early cardiac ischemic recovery^7^.

The role of *TREML4* in CAC was initially described by Sen et al.^8^ through an integrative analysis using omics technologies and suggested that *TREML4* functions as a modifier that plays a role in the conversion of soft plaque to calcified plaque rather than to be the cause of the presence of the plaque itself. These data support the hypothesis that the TREM Family locus, especially TREML4, may be responsible for various phenotypes in coronary artery disease and that gene expression can sometimes be related to different clinical outcomes.

Thus, in seeking to assess the *TREML4* expression profile throughout the progress of ischemic heart disease, our study aimed to investigate relationships between *TREML4* mRNA expression in blood leukocytes and its polymorphisms (rs2503495 and rs2803496) in asymptomatic patients and in patients who developed left ventricular (LV) dysfunction after MI.

## Results

### Clinical and biochemical laboratory data

Subclinical Atherosclerosis (SA) was detected in 60.88% of subjects (n = 207). Therefore, 39.12% (133) of the subjects did not present SA in the carotid doppler ultrasound (CDU) or Coronary tomography with calcium score (CAC) and were set as a control group. Clinical and biochemical laboratory data of SA are shown in Table 1. The mean age was higher in the SA group (*p* < 0.001). Patients with dyslipidemia and hypertension were more frequent in the SA group (*p* = 0.002 and *p* = 0.004, respectively) than controls. Laboratory data revealed that urea, creatinine, and tumor necrosis factor-alpha (TNF-α) significantly differed between groups (*p* = 0.015, *p* = 0.005, and 0.027, respectively). The use of medications in this sample is described in Supplementary Table 1. There was a higher usage frequency of statins (73.4%, *p* = 0.001) and diuretics (49.8%, *p* = 0.028) in SA patients.

**Table 1:** Clinical and laboratory data of subclinical Atherosclerosis and controls.

Variables	Control (133)	SA (207)	*p*-value
Age (years)	56 ± 7	61 ± 7	< 0.001
Male (%)	37.6 (50)	37.7 (78)	0.522
BMI (kg/m^2^)	29.9 ± 5.1	28.8 ± 4.8	0.076
Obesity (%)	36.1(48)	26.1 (54)	0.049
Dyslipidemia (%)	68.4 (91)	83.1 (172)	0.002
Diabetes (%)	14.3 (19)	18.8 (39)	0.276
Hypertension (%)	63.9 (85)	78.3 (162)	0.004
Metabolic syndrome (%)	73.7 (98)	77.8 (161)	0.387
Diastolic pressure (mmHg)	83 ± 12	82 ± 11	0.205
Systolic pressure (mmHg)	131 ± 20	133 ± 20	0.238
Physical activity (%)	59.8 (76)	53.1 (110)	0.231
Alcohol intake (%)	77.1 (101)	81.2 (168)	0.367
Smoking (%)	11.3 (15)	15.5 (32)	0.552
Family history of SAH (%)	60.2 (80)	55.1 (114)	0.356
Family history of DLP (%)	27.8 (37)	29.5 (61)	0.743
Family history of Diabetes (%)	48.9 (65)	38.2 (79)	0.051
Family history of Obesity (%)	13.5 (18)	9.2 (19)	0.208
Glucose (mg/dL)	104.9 ± 19.7	108.1 ± 26.5	0.161
HBA1c (%)	5.99 ± 0.63	6.13 ± 0.93	0.202
Insulin (uIU/mL)	12.15 ± 7.72	13.7 ± 20.47	0.936
Total cholesterol (mg/dL)	203.0 ± 42.0	197 ± 43.0	0.258
HDL cholesterol (mg/dL)	49.0 ± 14.0	47.0 ± 12.0	0.433
LDL cholesterol (mg/dL)	120.0 ± 37.0	115.0 ± 39.0	0.196
Triglycerides (mg/dL)	172.0 ± 106.0	176.0 ± 110.0	0.880
AST (U/L)	27.0 ± 11.0	27.0 ± 10.0	0.626
ALT (U/L)	37.0 ± 17.0	36.0 ± 16.0	0.517
Urea (mg/dL)	35.23 ± 9.30	37.34 ± 9.73	0.015
Creatinine (mg/dL)	0.89 ± 0.22	0.92 ± 0.20	0.076
Creatinine clearance (mL/min)	81.79 ± 21.22	76.74 ± 18.22	0.005
Microalbuminuria (mg/g)	45.03 ± 201.63	22.26 ± 62.52	0.204
Uric acid (mg/dL)	5.34 ± 1.35	5.44 ± 1.32	0.304
CPK (mg/dL)	122.0 ± 65.0	139.0 ± 99.0	0.876
hs-CRP (mg/dL)	4.82 ± 11.29	5.0 ± 14.72	0.440
Leptin (ng/mL)	22.68 ± 18.89	20.41 ± 17.58	0.299
Adiponectin (µg/mL)	30.49 ± 29.45	29.01 ± 25.67	0.855
Resistin (ng/mL)	9.03 ± 7.65	10.04 ± 10.18	0.857
TNF α (pg/mL)	5.09 ± 2.50	5.63 ± 2.58	0.027
PLA2 (ng/mL)	137.97 ± 50.81	145.5 ± 59.45	0.398
HOMA B (%)	120.17 ± 122.08	115.73 ± 157.66	0.485
HOMA IR (%)	3.16 ± 1.97	3.73 ± 5.74	0.958

Data are shown as the mean ± standard deviation or the percentage for categorical variables (number of patients). Parametric analysis was performed by ANOVA. Non-parametric samples were performed by Mann–Whitney U test. Categorical variables were compared by Chi-squared test. Control—Subjects without subclinical Atherosclerosis.

*AS* subclinical atherosclerosis patients, *SAH* systemic arterial hypertension, *DLP* dyslipidemia, *BMI* body mass index, *HDL* cholesterol high density lipoprotein, *LDL* cholesterol low density lipoprotein, *AST* aspartate aminotransferase, *ALT* alanine transaminase, *HBA1c* Hemoglobin A1c, *CPK* creatine phosphokinase, *hs-CRP* high-sensitivity C-reactive protein, *TNF α* tumour necrosis factor alpha, *PLA2* phospholipase A2, *HOMA B* homeostatic model assessment-beta-cell function, *HOMA* IR-homeostasis model assessment of insulin resistance.

All subjects with previous MI were matched for biodemographic characteristics, laboratory data, and some cardiac risk factors such as smoking, alcohol consumption, family history of MI, high blood pressure, and dyslipidemia, which were present in all of them (Table 2). Diabetes was a recurrent comorbidity in patients with LV dysfunction (*p* = 0.012) and consequently antidiabetic treatment (*p* = 0.008) (Supplementary Table 2).

**Table 2:** Clinical and laboratory data of patients according to LV dysfunction after MI*.*

Variables	LVEF > 40% (14)	LVEF ≤ 40% (14)	*p-*value
Female (%)	37.5 (5)	21.4 (3)	0.403
Age (years)	57.1 ± 7	60.8 ± 8.1	0.206
BMI (kg/m^2)^	28.1 ± 5.1	28.6 ± 4.6	0.795
Family history of MI (%)	71.4 (10)	71.4 (10)	0.999
Smoking (%)	64.3 (9)	50.0 (7)	0.445
Alcohol intake (%)	71.4 (10)	50.0 (7)	0.246
Physical activity (%)	71.4 (10)	71.4 (10)	0.999
Systolic pressure (mmHg)	120.0 ± 18.5	120.8 ± 14.7	0.598
Diastolic pressure (mmHg)	77.92 ± 8.91	80.0 ± 10.0	0.754
Time since last MI (months)	12.6 ± 24.1	47.9 ± 56.4	0.023
EF (%)	57.9 ± 7.5	32.2 ± 5.4	<0.001
Diabetes (%)	28.6 (4)	71.4 (10)	0.012
Obesity (%)	21.4 (3)	42.9 (6)	0.420
Glucose (mg/dL)	102.1 ± 17.2	124.3 ± 39.8	0.159
Total cholesterol (mg/dL)	156.6 ± 46.1	137.2 ± 29.5	0.598
HDL cholesterol (mg/dL)	36.17 ± 7.7	33.5 ± 10.8	0.488
LDL cholesterol (mg/dL)	89 ± 34.1	33.5 ± 10.8	0.413
Triglycerides (mg/dL)	171.3 ± 107.0	185.2 ± 116.4	0.597
Urea (mg/dL)	37.8 ± 11.3	57.9 ± 35.6	0.214
Creatinine (mg/dL)	0.9 ± 0.3	1.35 ± 0.7	0.101
ALT (U/L)	34.2 ± 18.35	33.7 ± 16.0	0.870
AST (U/L)	27.9 ± 13.0	27.0 ± 8.6	0.806

Data are shown as the mean ± standard deviation or the percentage for categorical variables (number of patients).

*n* number of individuals, *EF* ejection fraction, *BMI* body mass index, *MI* acute myocardial infarction, *HDL* high-density lipoprotein, *LDL* low-density lipoprotein, *ALT* alanine aminotransferase, *AST* aspartate aminotransferase.

### *TREML4* mRNA expression and polymorphisms

A total of 47.1% of SA (n = 160) and 64.3% of LV dysfunction (n = 9) subjects had *TREML4* expression values detected by quantitative reverse transcription (qRT)-polymerase chain reaction (PCR), whereas 52.9% and 35.7% tested positive for the *ACTB* gene alone, respectively. In addition, 50.0% of the SA (80) and 55.6% of the LV dysfunction subjects with positive *TREML4* on qRT-PCR had values above the group mean. No association was observed between *TREML4* expression and SA (*p* > 0.05) or LV dysfunction (Tables 3, 4).

**Table 3:** Relationship of *TREML4* mRNA expression with subclinical Atherosclerosis.

*TREML4* mRNA expression	Control	SA	*p*-value
	All (133)	All (207)	
Non-expression	52.6 (70)	53.1 (110)	0.927
Expression	47.4 (63)	46.9 (97)	
Low expression	50.8 (32)	49.5 (48)	0.871
High expression	49.2 (31)	50.5 (49)	

Number of subjects in parenthesis. Frequencies were compared by the Chi-Squared test. ‘SA’, subclinical Atherosclerosis; ‘Non-expression’, mRNA *TREML4* not expressed; ‘Expression’, mRNA *TREML4* expressed. ‘Low expression’, *TREML4* mRNA expression below the median value; ‘High expression’, *TREML4* mRNA expression above median value.

**Table 4:** Relationship of *TREML4* mRNA expression in the patients LV dysfunction after MI.

*TREML4* mRNA expression	LVEF > 40% All (14)	LVEF ≤ 40% All (14)	*p*-value
Non-expression	49.2 (6)	35.7 (5)	0.699
Expression	57.1 (8)	64.3 (9)	
Low expression	75.0 (6)	44.4 (4)	0.201
High expression	25.0 (2)	55.6 (5)	

Number of subjects in parenthesis. Frequencies were compared by the Chi-Squared test. ‘MI’, acute myocardial infarction; ‘Non-expression’, mRNA *TREML4* not expressed; ‘Expression’, mRNA *TREML4* expressed. ‘Low expression’, *TREML4* mRNA expression below the median value; ‘High expression’, *TREML4* mRNA expression above median value.

The genotype and allele frequencies were similar between the SA and Control groups (*p* > 0.05, Table 5). The genotype frequencies of *TREML4* polymorphisms (rs2803495 and rs2803496) were in Hardy–Weinberg equilibrium, indicating no genotyping errors in breeding and evolutionary pressure. Linkage disequilibrium between rs2803495 and rs2803496 was not detected (D′ = 0.99, *p* = 0.006).

**Table 5:** Relationship of *TREML4* polymorphisms with subclinical Atherosclerosis (SA).

Polymorphism			Control	SA	*p*-value
rs2803495 A/G		N = 325	N = 127	N = 198	
Genotypes (%)	AA	82.2 (267)	82.7 (105)	81.8 (162)	0.844
(Codominant)	AG	17.8 (58)	17.3 (22)	18.2 (36)	
	GG	0 (0)	0	0	
(Dominant)	AA	82.2 (267)	82.7 (105)	81.8 (162)	0.844
	AG + GG	17.8 (58)	17.3 (22)	18.2 (36)	
Alleles (%)	A	100 (325)	100 (125)	100 (198)	0.911
	G	17.8 (58)	17.3 (22)	18.2 (36)	

rs2803496 C/T		N = 324	N = 125	N = 199	
Genotypes (%)	TT	82.2 (262)	83.2 (104)	79.4 (158)	0.369
(Codominant)	CT	17.0 (55)	16.0 (20)	17.6 (35)	
	CC	2.2 (7)	0.8 (1)	3.0 (6)	
(Dominant)	TT	80.9 (262)	83.2 (104)	79.4 (158)	0.397
	CT + CC	19.1 (62)	16.8 (21)	20.6 (41)	
Alleles (%)	T	97.83 (317)	99.2 (124)	96.9 (193)	0.437
	C	19.13 (62)	16.8 (21)	20.60 (41)	

Number of subjects in parenthesis. Frequencies were compared by the Chi-Squared test. *p*-values refer to comparisons among genotype frequencies of the different models and among allelic frequencies. < 10% of all samples did not have their genotype detected. SA-Subclinical Atherosclerosis.

We previously described that not every patient with clinical Atherosclerosis could express *TREML4* in blood leukocytes, and this expression is associated with the presence of the rs2803496 C allele (TC/CC genotypes)^6^. In this cohort, the genotype rs2803495 (AG) was more frequent (31.2%, *p* < 0.001) in the group that showed TREML4 expression than the one that did not express this gene (Table 6). Genotypes (CT + CC) were also more frequent (34.4%, *p* < 0.001) for rs2803496 in the TREML4 expressing group (Table 6). Subjects with the G allele (2,803,495) were more likely to express TREML4 (OR 8.01, 95% CI 3.78–16.99, *p* < 0.001). The C allele (rs2803496) also confers greater chances of expressing TREML4 (OR 10.42, 95% CI 4.76–22.78, *p* < 0.001). Moreover, subjects with the C allele were more likely to have high TREML4 mRNA expression (OR 4.88, 95% CI 2.35–10.12, *p* < 0.001) (Table 7).

**Table 6:** Relationship of polymorphisms with *TREML4* mRNA expression.

Polymorphism		*TREML4* mRNA expression					*p*-value
		Non-expression	Expression	*p*-value	Low	High	
rs2803495 A/G		N = 168	N = 157		N = 79	N = 78	
Genotypes (%)	AA	94.6 (159)	68.8 (108)	** < *0.001***	65.8 (52)	71.8 (56)	*0.419*
(Codominant)	AG	5.4 (9)	31.2 (49)		34.2 (27)	28.2 (22)	
	GG	0.0 (0)	0.0. (0)		0.0 (0)	0.0 (0)	
(Dominant)	AA	94.6 (159)	68.8 (108)	** < *0.001***	65.8 (52)	71.8 (56)	*0.419*
	AG + GG	5.4 (9)	31.2 (49)		34.2 (79)	28.2 (22)	
Alleles (%)	A	100.0 (168)	100 (157)	** < *0.001***	100.0 (79)	100 (78)	*0.558*
	G	5.4 (9)	31.2 (49)		34.2 (27)	28.2 (22)	

rs2803496 C/T		N = 167	N = 157		N = 79	N = 78	
Genotypes (%)	TT	95.2 (159)	65.6 (103)	** < *0.001***	82.3 (65)	48.7 (38)	** < *0.001***
(Codominant)	CT	4.2 (7)	30.6 (48)		15.2 (12)	46.2 (36)	
	CC	0.6 (1)	0.6 (6)		2.5 (2)	5.1 (4)	
(Dominant)	TT	95.2 (159)	65.6 (103)	** < *0.001***	82.3 (65)	48.7 (38)	** < *0.001***
	CT + CC	4.8 (8)	34.4 (54)		17.7 (14)	51.3 (40)	
Alleles (%)	T	99.4 (166)	96.17 (151)	** < *0.001***	97.4 (77)	94.8 (74)	***0.0014***
	C	4.79 (8)	34.39 (54)		17.7 (14)	51.3 (40)	

Number of subjects in parenthesis. Frequencies were compared by the Chi-Squared test. *p*-values refer to comparisons among genotype frequencies of the different models and among allelic frequencies. < 10% of all samples did not have their genotype detected. ‘Non-expression’, mRNA *TREML4* not expressed; ‘Expression’, mRNA *TREML4* expressed. ‘Low expression’, *TREML4* mRNA expression below the median value; ‘High expression’, *TREML4* mRNA expression above median value.

Significant values are in bolditalics.

**Table 7:** Relationships of *TREML4* polymorphisms, rs2803495 allele G and rs2803496 allele C with *TREML4* mRNA expression groups.

Model	Variables	B	OR (IC 95%)	*p*-value
1	rs2803495 (G allele)	2.08	8.01(3.78–16.99)	** < 0.001**
2	rs2803496 (C allele)	2.34	10.42 (4.76–22.78)	** < 0.001**
3	rs2803495 (G allele)	− 0.27	0.75(0.38–1.49)	0.420
4	rs2803496 (C allele)	1.58	4.88(2.35–10.12)	** < 0.001**

Model 1: Univariate logistic regression analysis. Dependent variable: *TREML4* mRNA expression, non-expression and *TREML4* expression. Independent variable: rs2803495 allele G, yes/no. Model 2: Univariate logistic regression analysis. Dependent variable: *TREML4* mRNA expression. Independent variable: rs2803496 alle C, yes/no. Model 3: Univariate logistic regression analysis. Dependent variable: *TREML4* mRNA expression below median value/above median value. Independent variable: rs2803495 allele G, yes/no. Model 4 Univariate logistic regression analysis. Dependent variable: *TREML4* mRNA expression below median value/above median value. Independent variable: rs2803496 allele C, yes/no.

*OR* odds ratio, *CI* confidence interval.

Significant values are in bold.

## Discussion

Herein we report for the first time that *TREML4* is not associated with the early stages of atherosclerotic plaque formation and later stages after MI. In a previous study, we analyzed the expression of *TREML4* and its polymorphisms in blood leukocytes from 137 patients with coronary artery disease. We observed that patients with higher levels of atherosclerotic lesions were associated with higher *TREML4* expression levels and that this expression is influenced by the presence of the rs2803495 and rs2803496 variants^6^. Furthermore, similar to our previous findings, only some associations between classic risk factors with subclinical Atherosclerosis and heart failure post-MI were observed in our study, as shown in Tables 1, 2. Together, our observations support the hypothesis that classical risk factors for cardiovascular diseases are not sensitive enough to assess atherosclerotic lesions or to stimulate coronary lesions and strengthen the importance of new, more sensitive, and minimally invasive biomarkers to assess the presence and burden of atherosclerotic lesions.

Thus, we also observed that *TREML4* is more expressed during acute events such as ACS through a transcriptomic study using a microarray model and validated by a real-time polymerase chain reaction in patients with ACS^7^. Similarly, it was possible to observe that the presence of minor alleles of polymorphisms rs2803495 (G) and rs2803496 (C) confer greater cardiovascular risk and may favor the expression of *TREML4* through two cis-eQTL single nucleotide polymorphism (SNP) analyses based on a multi-omics analysis in patients with coronary artery calcification^8^.

Through transcriptomic analysis in human and murine macrophages, Gonzalez-Cotto et al.^17^ recently demonstrated that *TREML4* is more preferentially expressed in inflammatory macrophages and favors disease development by increasing the lesion load and macrophage content. Their transcriptome results report that the presence of lower frequency alleles can increase *TREML4* expression up to 300-fold. In order to obtain more information about the expression profile of genes possibly controlled by *TREML4* expression, the same group performed RNA sequencing analysis in human blood macrophages that were carriers of haplotypes considered permissive (carriers of the minor alleles G and C) for *TREML4* expression and observed that TREML4 regulates genes from several pathways, primarily related to the inflammatory response (*TLR4*^10^*, TLR13*^11^*, MMP25*^12^*, RUNX3*^13^, lipid regulation *EHD1*^14^*, LIPG*^15^*, ABCA5*^16^ and carbohydrate metabolism^17^.

In a previous study, we demonstrated that *TREML4* seems to be associated with the regulation of pathways involving carbohydrate metabolism and that patients who have Diabetes mellitus type 2 and are carriers of the minor allele (c) (rs2803496) may have an increase of up to 20.9 times in the expression of *TREML4*^8^. Interestingly, a recent study analyzed the transcriptional profile of genes in Apoe −/− /Treml4 −/− mice Macrophages and demonstrated that genes in the Glycolysis/gluconeogenesis pathways are regulated by *TREML4*^17^.

**Figure 1:**
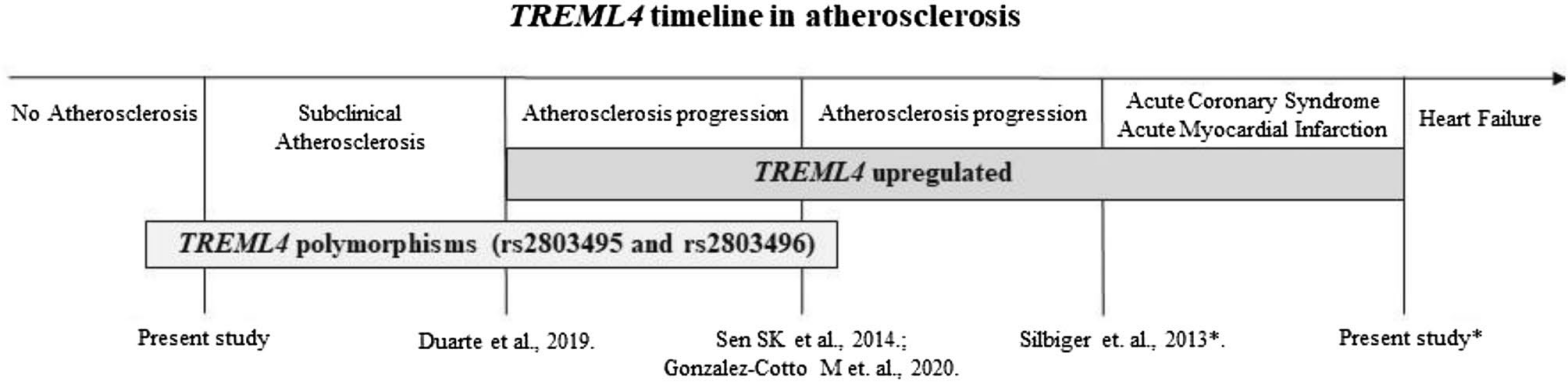
*TREML4 *and polymorphism timeline in the progress of Atherosclerosis. *SNPs were not investigated in these sample.

Our current and previous results show that the transcriptional profile of TREML4 is present between the initial to intermediate stages of atherosclerotic disease progression, as illustrated in Fig. 1. As it is a multifactorial and inflammatory disease, more advanced stages of this disease are characterized by the presence of an inflammatory microenvironment^18^, with the presence of calcification^8^ which commonly progresses to ACS^7^, and its advance is influenced by the presence of polymorphisms ^19^.

Of the subjects with SA in our study, 18.2% have the rs2803495 AG + GG genotype, and 20.6% have the rs2803496 CT + CC genotype. These results are similar to those in a European population in phase 3 of the 1000 Genomes project (http://www.internationalgenome.org/). Additionally, we observed that subjects who carry the minor alleles G (AG + GG) or C (CT + CC) are more likely to express *TREML4.* SNP can impact mRNA in several ways, whether in mRNA splicing, nucleo-cytoplasmic export, stability, or translation^20^. rs2803495 and rs2803496 are variants in the regulatory region and the 5'-untranslated region (UTR) of chromosome 6^21^. The 5’UTR regions contain key elements in transcriptional and translational regulation, such as the upstream open reading frames (uORFs), which can control the selection of translation-initiation sites or even contribute to the translatability of mRNA^22^, which may precisely impact the regulation of *TREML4* expression. Although we did not find a relationship between SNPs and TREML4 with SA (early stage) and LV dysfunction after MI (a final consequence of MI), we hypothesize that TREML4 polymorphisms, expression, and other regulatory molecules such as microRNAs, may be critical to compress the progression of atherosclerotic burden^6^ and the rupture plate^7^.

## Conclusion

In conclusion, this study evaluated the relationship between gene expression and TREML4 polymorphisms in patients with subclinical atherosclerosis compared to a control group and LV dysfunction after MI. Our results in this cohort and our previous results^6,7^ suggest that genetic polymorphisms in TREML4, including in asymptomatic patients, influence mRNA expression in leukocytes and may regulate differently during the atherosclerosis progression stages, but not in symptomatic Atherosclerosis disease and post-MI.

## Methods

### Subclinical atherosclerosis sample

A total of 340 subjects who had subclinical atherosclerosis (SA) as verified in their hospital records were enrolled for screening in this cross-sectional study between February 2010 and March 2013. The patients were recruited during their routine appointment at the Medical Sections of Dyslipidemia, Arterial Hypertension, and Nephrology of the Dante Pazzanese Cardiology Institute (DPCI), São Paulo, SP, Brazil.

The clinical outcome of the SA patients was according to protocols presented by Bertolami et al.^23^. Carotid Doppler ultrasound (CDU) and Coronary tomography with calcium score (CAC) were used to detect SA. Subjects with CAC > 0 or the presence of plaque and/or increased intima thickness above the 75th percentile for age, gender and race in the CDU according to the criteria of the MESA study were included in the SA group. The control group consisted of individuals who did not present SA in any of the methods.

The Research Ethics Committee of DPCI approved the study of this sample, which complies with the Declaration of Helsinki, under protocol number 3852/2009. Written informed consent was obtained from each participant before sample collection, and all experiments were performed following relevant guidelines and regulations. Clinical data were assessed from each patient in their hospital records.

### Heart failure post-MI sample

A total of 65 subjects who had suffered previous STEMI at least two months prior to inclusion in the study as verified in their hospital records were enrolled for screening in this cross-sectional study between July 2018 and December 2019. The patients were recruited during their routine appointment at the Cardiology Outpatient Unit, Onofre Lopes University Hospital of the Federal University of Rio Grande do Norte (UFRN), Natal, RN, Brazil.

The clinical outcome of the STEMI patients was determined by LVEF measured at ± 3 months from the date of venous blood collection. Since ejection fraction is widely used to determine LV dysfunction, patients were classified into two groups: LV dysfunction and symptoms of HF (LVEF ≤ 40%, n = 14) and those with normal LV function (LVEF > 40%, n = 14), according to the American Heart Association Guideline for the Management of HF^17^. The exclusion criteria included other cardiac diseases that could lead to HF such as congenital cardiomyopathies, dilated cardiomyopathy, atrial fibrillation, Chagas disease, and non-treated hypertension; patients forwarded to cardiac transplants were not enrolled.

The Research Ethics Committee of UFRN approved the study of this sample, which complies with the Declaration of Helsinki under protocol number 2.017.026. Written informed consent was obtained from each participant before sample collection, and all experiments were performed following relevant guidelines and regulations. Clinical data were assessed from each patient in their hospital records.

### Biochemical measurements

Fasting serum glucose, triglycerides, total cholesterol, high-density lipoprotein (HDL)-cholesterol, Urea, creatinine, uric acid, alanine aminotransferase, and aspartate aminotransferase (AST) were measured using colorimetric and enzymatic colorimetric assays. Low-density lipoprotein (LDL)-cholesterol levels were calculated according to the Friedewald formula. The CKD-EPI equation obtained creatinine clearance. High sensitivity C-reactive protein (hs-CRP) was analyzed by Nephelometry. CPK measurements were performed using the modified Szasz method. The high-performance liquid chromatography (HPLC) method was used to measure HbA1c. Insulin was performed by chemiluminescence. A “sandwich”-type enzyme immunoassay was performed using the CHEMICON® Adiponectin Sandwich ELISA Kit (Chemicon International, CA, USA). TNFα and Lp-PLA2 were measured by Luminex™ xMAP for resistin, and radioimmunoassay was used for leptin.

Insulin resistance was evaluated using the Homeostasis Model Assessment Insulin Resistance (HOMA-IR)^11^, calculated by applying the formula: (FG*fasting insulin)/405. Beta-cell function was evaluated utilizing Homeostasis Model Assessment Beta (HOMA-B) by the formula: (20 × fasting insulin)/[(FG/18) − 3.5].

### RNA isolation and quantitative polymerase chain reaction (qPCR) analysis

Total RNA was extracted from leukocytes stored in RNAlater® stabilization solution (Life Technologies, Carlsbad, CA, USA) using a RiboPureTM Blood kit (Life Technologies) for SA sample and Trizol protocol (Invitrogen, Massachusetts, USA) for HF. RNA integrity was assessed by the TapeStation® system (Agilent Technologies, Santa Clara, USA) or gel with GelRed (Uniscience, São Paulo, SP, Brazil), revealing the presence of two sharp bands at approximately 5 and 1.8 Kb, corresponding to 28S and 18S ribosomal RNA, respectively. RNA concentration and purity (260/280) were measured using a Qubit® 2.0 Fluorometer (Life Technologies) and NanoDrop® ND1000 spectrophotometer (Thermo Fisher Scientific Inc., Waltham, USA), respectively. RNA samples were stored at − 80 °C.

mRNA expression was analyzed by qRT-PCR. cDNA was synthesized using a High-Capacity cDNA Reverse Transcription Kit (Applied Biosystems, Foster City, CA, USA) in a MyCycler Thermal Cycler (Bio-Rad, Hercules, CA, USA). qPCRs were run using TaqMan assay (*TREML4*, Hs01080584_g1; Life Technologies). The reference gene was selected from a normalization study of three endogenous candidate genes: *GAPDH* (Hs.592355_g1), *ACTB* (Hs.520640_g1), and *18S rRNA* (Hs.626362_g1) using geNorm and NormFinder software. qPCRs were run in the Rotor-Gene Q Real-Time PCR Detection System (QIAGEN GmbH, Hilden, Germany) for SA, and 7500 Fast Real-Time PCR instrument (Applied Biosystems, CA, USA) for HF. Relative mRNA expression was calculated using the 2–ΔCT method, with *ACTB* as a reference gene.

### DNA isolation and genotyping

According to the manufacturer’s instructions, the genomic DNA was isolated from whole blood collected in EDTA tubes using the QIAamp DNA Blood Mini Kit (Qiagen, Hilden, Germany). DNA quantification was measured by a UBIT® 2.0 fluorometer (Life Technologies, Forest City, USA). Purity (260/280) was performed using a NanoDrop® ND1000 spectrophotometer (Thermo Fisher Scientific Inc., Waltham, USA) and Integrity 2200 TapeStation® system (Agilent Technologies, Santa Clara, USA). DNA samples were stored at − 20 °C until analysis. *TREML4* polymorphisms rs2803495 (A > G) and rs2803496 (C > T) were genotyped by qRT-PCR using TaqMan SNP Genotyping Assays (C_27302616_10 and C_27302614_10) (Life Technologies) in a Rotor-Gene Q Real-Time PCR Detection System (QIAGEN GmbH, Hilden, Germany), according to the manufacturer’s protocol. Ten percent of randomly selected DNA samples were assayed in duplicate, and SNPs were 100% confirmed and concordant in the duplicate pairs. Genotyping analysis was not performed for the patients who had suffered previous MI.

### Statistical analysis

Statistical analysis was performed using the SPSS® 22.0 software program (SPSS, Inc., Chicago, IL, USA) (www.ibm.com/br-pt/products/spss-statistics). Normal distribution was evaluated using the Kolmogorov–Smirnov test. Continuous variables with normal distributions are presented as the mean and standard deviation and were compared using t-tests or analysis of variance followed by Tukey’s test. Variables without parametric distributions are presented as the median and were analyzed using the Kruskal–Wallis test followed by the Mann–Whitney test. The chi-squared test compared categorical variables. Independent variables possibly affecting *TREML4* mRNA expression were determined by univariate logistic regression analysis. A *p*-value < 0.05 was considered significant. Linkage disequilibrium was evaluated using the HAPLOVIEW® 4.2 software program (www.broadinstitute.org/haploview/haploview)^24^.

## Supplementary Information

Supplementary Information.

## Data Availability

All data generated or analysed during this study are included in this published article (and its Supplementary Information files).
