# Supplementary material for: TREML4 polymorphisms increase the mRNA in blood leukocytes in the progression of atherosclerosis
Source: Sci Rep. 2022 Nov 3;12:18612. doi: 10.1038/s41598-022-22040-3 (PMC9633690; doi:10.1038/s41598-022-22040-3)
Supplement: Supplementary file 1 — Supplementary Information. [file 41598_2022_22040_MOESM1_ESM.pdf]

TREML4 polymorphisms increase the mRNA in blood leukocytes in the progression of atherosclerosis.

Victor Hugo Rezende Duarte[a]*, Marina Sampaio Cruz[a], Adriana Bertolami[b], Mario Hiroyuki Hirata[c], Rosario Dominguez Crespo Hirata[c], André Ducati Luchessi[a] & Vivian Nogueira Silbiger[a]*.

[a]Department of Clinical and Toxicological Analyses, Federal University of Rio Grande do Norte. General Cordeiro de Farias Av., Natal, Rio Grande do Norte, 59012-570, Brazil,

[b]Dyslipidemia Medical Section, Dante Pazzanese Institute of Cardiology, Av. Dr. Dante Pazzanese, 500, 04012-909, São Paulo, Brazil.

[c]Department of Clinical and Toxicological Analyses, School of Pharmaceutical Sciences, University of Sao Paulo. 580 B17 Lineu Prestes Av., Butantan,05508-900. Sao Paulo, Brazil.

*Corresponding authors

E-mail address; V.H.R.D (email: victorhugorezendeduarte@gmail.com)

and V.N.S. (email: vivian.silbiger@ufrn.br)

Address: Department of Clinical and Toxicological Analysis, Federal University of RioGrande do Norte, Natal, Brazil. Avenue General Gustavo Cordeiro de Farias, S/N, Natal, Rio Grande do Norte CEP: 59014-520, Brazil

Supplementary table 1. Use of medications in subclinical Atherosclerosis and controls.

Medications	Control (133)	SA (207)	*p-value*
Ezetimibe, %	6.80 (9)	12.1 (25)	0.111
Statins, %	55.6 (74)	73.4 (152)	0.001
Diuretics, %	37.6 (50)	49.8 (103)	0.028
ACEIs, %	39.1 (52)	49.6 (97)	0.159
Vasodilators, %	0.8 (1)	1.9 (4)	0.378
Fibrate, %	5.3 (7)	2.4 (5)	0.165
Beta-Blockers, %	30.1 (40)	30.0 (62)	0.981
ASA, %	33.1 (44)	40.1 (83)	0.192
Spironolactone, %	3.8 (5)	3.9 (8)	0.961

Data are shown as the percentage and number of patients in parentheses. The variables were compared by the Chi-squared test. ACEIS - angiotensin-converting enzyme inhibitors, ASA - acetylsalicylic acid.

Supplementary Table 2. Use of medications by patients with LV dysfunction after MI.

Medications	LVEF > 40% (14)	LVEF ≤ 40% (14)	*p-value*
Antihypertensive, %	100 (14)	100 (14)	0.999
Statins, %	100 (14)	100 (14)	0.999
Platelet aggregation inhibitors, %	100 (14)	100 (14)	0.999
Diuretics, %	28.6 (4)	71.4 (10)	0.023
Antidiabetics, %	21.4 (3)	71.4 (10)	0.008

Data are shown as the percentage and number of patients in parentheses. The Chi-squared test compared the variables.
